# Intention to Stop Bullying following a Condemning, Empathy-Raising, or Combined Message from a Teacher – Do Students’ Empathy and Callous-Unemotional Traits Matter?

**DOI:** 10.1007/s10964-022-01613-5

**Published:** 2022-04-16

**Authors:** Eerika Johander, Jessica Trach, Tiina Turunen, Claire F. Garandeau, Christina Salmivalli

**Affiliations:** 1 INVEST Flagship Research Center/ Department of Psychology and Speech-Language Pathology, University of Turku, Turku, Finland; 2 Shandong Normal University, Jinan, China

**Keywords:** Bullying, Targeted interventions, Teacher messages, Empathy, Callous-unemotional traits, Experimental design

## Abstract

Knowing which intervention strategies work best and for which student is essential for teachers when they intervene in cases of bullying. The effects of teachers’ (1) condemning, (2) empathy-raising, and (3) combined (including elements of both) messages on students’ intention to stop bullying were tested in a between-subject experimental design. A total of 277 seventh grade students (*M*_age_ = 12.93, SD = 0.49; 47% female) were asked to imagine they had bullied a peer and were invited to a discussion with a teacher. They saw a video vignette with one of the above messages. Hierarchical regression analyses indicated that students’ intention to stop bullying was highest among those who saw the combined message. Callous-unemotional traits were negatively, and affective and cognitive empathy positively associated with intention to stop bullying. Students’ level of cognitive empathy moderated the relative effect of the condemning message on intention to stop bullying. At low levels of cognitive empathy, the condemning message was the least effective, whereas among those with high cognitive empathy, all messages were equally likely to lead to intention to stop bullying. Together, the findings suggest that for educators intervening in bullying among adolescents, an approach involving both condemning and empathy-raising messages is the ‘best bet’, most likely to lead to intention to stop bullying.

## Introduction

Teachers and other school personnel have the responsibility to intervene quickly when a case of bullying comes to their attention. Knowing which intervention strategies work best and for which student is essential for them. The few studies that have examined the relative effectiveness of different targeted anti-bullying interventions in real-life settings (Garandeau et al.,^ 2014^b; Garandeau et al., 2016; Johander et al., 2021) are limited in two important ways. First, despite providing instructions to teachers about how to respond to bullying situations, the extent to which teachers followed these instructions was not known and could not be controlled for in the studies. Second, the potential moderating effects of psychological characteristics of the students were not investigated. To fill the gaps, the present study uses an experimental design with video vignettes that depict a teacher talking to a student (the participant) after they have hypothetically bullied a peer. This design allows the manipulation of the type of message used by the teacher (condemning the bullying behavior, raising empathy for the victim, or both), so that the exact content of the message is known, and the effects of different strategies can be directly compared. The study examines the effects of the message on students’ intention to stop bullying. In addition, this study examines whether students’ responses to the different messages vary as a function of their levels of empathy and callous-unemotional traits, which have been shown to be related to bullying behavior (Geel et al., 2017; Mitsopoulou & Giovazolias, 2015). As anti-bullying interventions tend to become less effective in adolescence (e.g., Salmivalli et al., 2021; Yeager et al., 2015), it is important to understand students’ responsiveness to various intervention strategies at this age. For this reason, the present experiment was conducted with early adolescents in their first year of secondary school.

### Effectiveness of Confronting and Non-Confronting Approach

Studies on interventions targeted at bullying perpetrators have investigated two major approaches: a direct, confronting approach, and an indirect, non-confronting approach (e.g., Garandeau et al., 2014b). The confronting approach consists in telling the perpetrator that the adults at school know about their bullying behavior, that it is not tolerated, and must stop immediately (Olweus, 1993). This approach focuses on setting clear and firm limits for behavior. The non-confronting approach, originally derived from the Method of Shared Concern (Pikas, 1989) and the Support Group Method (Robinson & Maines, 2008), aims to increase the perpetrator’s empathy for their victim without accusing them of any wrongdoing. Instead, the adult shares his or her concern about the difficulties experienced by the victimized peer, without taking a stand on who is responsible for this painful situation. The main goal is to get the perpetrator to share the adult’s concern for the victimized peer and provide solutions to improve the situation (i.e., engage in theory-of-mind type of behavior where they hopefully increase their empathic concern).

To date, only three studies have directly compared the confronting and non-confronting approach. Two studies examined their short-term effectiveness in the context of the randomized controlled trial (RCT) of the KiVa antibullying program in Finland (Garandeau et al., 2014b; Garandeau et al., 2016), and one examined their long-term effectiveness after the nationwide roll-out of the same program (Johander et al., 2021). In the RCT, half of the intervention schools were instructed and trained to use the confronting approach, whereas the other half were instructed and trained to use the non-confronting approach; however, the exact content of the discussions was not observed or recorded. First, the effectiveness of the approaches was evaluated by asking the victims in a follow-up meeting, about 2 weeks after the intervention, whether the bullying had stopped (Garandeau et al.,^ 2014^b). According to the victimized students, bullying had stopped in 78.2% of the cases. After controlling for the level of schooling (primary versus secondary school), type of aggression (e.g., verbal, physical, relational, online), and the duration of victimization (how long the bullying had been going on), neither approach was shown to be overall more effective than the other. However, some factors were found to moderate the relative effectiveness of the approaches. Although the two approaches were equally successful in cases of long-term victimization (more than 6 months) and in primary schools (Grades 1–6), the confronting approach was slightly more successful than the non-confronting approach when the bullying had been happening for less than 6 months, or took place among secondary school students (Grades 7–9).

Second, the effectiveness of the approaches was studied by examining how bullying perpetrators’ perceptions of the targeted interventions influenced their intention to change their behavior (Garandeau et al., 2016). Right after meeting with a teacher to discuss their behavior, students who were bullying others were invited to report in an anonymous questionnaire the extent to which they perceived that the teacher had (a) condemned their behavior, and (b) tried to arouse their empathy for the victimized peer. Bullies’ intention to change their behavior (i.e., stop bullying) was overall quite high (mean of 4.12 on a scale from 0 to 5). Perceiving that the teacher had condemned the bullying behavior and perceiving that the teacher had tried to raise their empathy for the victim both had a positive – and equally strong – effect on bullies’ intention to change their behavior. In addition, their intention to change behavior was highest when they felt that the teacher had both condemned the bullying behavior *and* tried to arouse their empathy for the victim, rather than using only one of the two strategies.

Finally, whereas previous studies had used reports from single informants—victimized children or perpetrators—collected shortly after the intervention discussion, a recent study examined the long-term effectiveness of the same approaches using reports from both school personnel and victimized students (Johander et al., 2021). The data were collected annually across six years via online questionnaires and included responses from students and personnel in 1221 primary and secondary schools. At the end of each school year, the school personnel were asked to indicate which approach they had used in handling cases of bullying during the past school year and to evaluate the effectiveness of their interventions. Similarly, students who reported that they had been victimized and that the bullying had been addressed by the adults at school, were asked whether the interventions had an effect on their situation. Overall, the discussions were found to be quite effective in reducing bullying; according to victimized students, the bullying had decreased or stopped in 74 % of the cases and the mean of personnel-perceived effectiveness of the discussions was 3.17 (on a scale from 0–4, where a value of 3 indicated that the discussions had been rather successful and 4 meaning that they had been very successful). Importantly, the effectiveness of the discussions did not vary depending on whether the school personnel reported that they consistently used the confronting or the non-confronting approach across cases, or whether they varied strategy depending on the situation. However, the discussions were significantly *less* effective (according to both students and school personnel) when the personnel reported that they had used their own adaptations rather than followed the program-recommended, evidence-based approaches.

An important limitation of previous studies examining the effectiveness of confronting and non-confronting approach is that the exact content of the adult discussions with the bullies could not be verified. The studies either relied on what teachers were instructed to do, what they reported doing, or what students reported happened during the discussion. These reports were therefore subjective, based on retrospective accounts, or depended on whether the approaches were implemented as instructed. The current study addresses these limitations by using an experimental design where the message delivered by the teacher was known, and the effects of different messages on students’ intentions to stop bullying could be directly compared.

### Individual Student Characteristics as Possible Moderators of Effectiveness of Confronting and Non-Confronting Approaches

#### Empathy

Another important limitation of the previous research is the lack of information on how psychological characteristics of the students’ receiving the intervention might moderate their response to these various approaches. One personal characteristic that likely influences the effectiveness of the different approaches considered in this study is empathy, which refers to the ability to feel or imagine another person’s emotions and is often divided into affective and cognitive components (Cuff et al., 2016). Affective empathy is defined as the ability to *experience* the feeling of another person, and cognitive empathy as the ability to *understand* the emotions and perspective of others. The non-confronting approach focuses on arousing the perpetrators’ empathy for their victims. Thus, the expected change in bullying behavior is reliant on the perpetrators’ capacity to feel empathy. However, there is clear evidence that children and adolescents who bully others tend to be deficient in empathy, especially affective empathy (van Noorden et al., 2015; Zych & Llorent, 2019). This could imply that attempts at stopping bullying behavior by trying to appeal to perpetrators’ capacity to feel empathy are unlikely to work with bullies low on affective empathy. Findings regarding the association between bullying and cognitive empathy are less consistent. Although the association is generally found to be negative (e.g., van Noorden et al., 2015), its magnitude is quite small (Mitsopoulou & Giovazolias, 2015). Thus, it is also possible that youth who bully others are already aware of the suffering their behavior is causing for the victim. Therefore, merely telling the perpetrator what they already know (i.e., their behavior makes the victimized peer suffer) might be unproductive.

#### Callous-unemotional traits

In addition to empathy, callous-unemotional traits may also influence the effectiveness of the different approaches. Individuals high in callous-unemotional traits are characterized not only by a lack of empathy, but especially by a lack of guilt and remorse, as well as shallow or deficient affect (Frick, 2009; Kimonis et al., 2015). Callous-unemotional traits are also positively associated with different forms of antisocial behavior, including bullying (Frick & White, 2008; Zych et al., 2019). Some researchers have suggested that anti-bullying programs should aim to reduce callous-unemotional traits among youth by increasing their overall empathic concern for the victim and utilizing authoritative school discipline and teacher style (including warmth, promotion of autonomy, enforcement of rules and standards, use of reprimands and punitive strategies when necessary; Ertesvåg, 2011; Thornberg & Jungert, 2017). However, others suggest that neither approaches that rely on raising empathy for the victim nor punitive strategies involving exclusion from school and other highly disciplinary sanctions are likely to be successful with youth high in callous-unemotional traits because these individuals have evidenced difficulties with empathy and reduced responsivity to punishments in learning new strategies (Blair et al., 2006; Viding et al., 2009; Waller et al., 2020). Although the confronting approach does not include sanctions (other than the discussion itself), it is a disciplinary strategy. Thus, it might be that attempts at stopping bullying by condemning the bullying behavior are unlikely to be successful at changing the behavior of youth who are high in callous-unemotional traits.

Moreover, children and adolescents high in callous-unemotional traits have been found to have impairments in functions required for empathic reactions, such as recognition of cues of sadness and fear in others (for reviews, see Frick et al., 2014; Frick & White, 2008). In addition, youth high in such traits show deficits in activation of brain areas involved in the processing of emotional stimuli, such as reduced amygdala responses to fearful expressions (Marsh, 2008; Viding et al., 2012). Thus, it seems that youth high in callous-unemotional traits might lack the capacity for empathic responding. As the non-confronting approach is based on empathy induction strategies, it could be expected to be less effective among youth with high levels of callous-unemotional traits.

Nevertheless, studies conducted with youth low on empathy or high on callous-unemotional traits have shown that such populations could respond positively to empathy-inducing interventions. For example, the Finnish KiVa antibullying program was found to have a positive effect on affective empathy and these effects were independent of the students’ initial levels of empathy (Garandeau et al., 2021). Similarly, a training designed to facilitate emotion recognition in children with different behavioral and emotional problems was found to increase affective empathy and decrease conduct problems in children high on callous-unemotional traits (Dadds et al., 2012). In another study with experimental design, children with 10–11 years of age were asked to play a competitive computer-based game against a virtual opponent, where they had the opportunity to blast a noise into the headphones of the other ‘player’ (Van Baardewijk et al., 2009). The intensity of the noise they chose to blast at their opponent was used as an indicator of their aggression. Children higher in psychopathic traits were more aggressive towards their opponent, except when the distress of the target was made salient through a written message expressing his or her fear. In the empathy-raising message, the distress of the victimized student is expressed to the perpetrator verbally by the adult carrying out the discussion. Two of these studies examined the effects of long-term program implementation (Garandeau et al., 2021) or training (Dadds et al., 2012) rather than immediate reactions (Van Baardewijk et al., 2009) or short-term interventions; however, they all suggest that even students low in empathy can be expected to change their behavior in response to the empathy-raising message.

## Current Study

Using a between-subject experimental design, this study investigates early adolescents’ intention to stop (hypothetical) bullying after watching a video in which an adult is talking to them after they have supposedly bullied a peer, using three different messages: condemning (as in the confronting approach), empathy-raising (as in the non-confronting approach), or a combination of both. In addition to examining students’ responses to these three experimental conditions, this study tests whether students’ empathy and callous-unemotional traits moderate the relative effectiveness of the different messages. First, it is hypothesized that viewing the combined message will be associated with higher intention to stop bullying than viewing the condemning only or the empathy-raising only message. Based on prior findings, no significant difference between the effects of the condemning message and the effects of the empathy-raising message is expected. Second, it is hypothesized that both types of empathy will be positively associated and callous-unemotional traits will be negatively associated with intention to stop bullying. Third, it is hypothesized that the empathy-raising and the combined message would work better than the condemning message among those who are high on empathy compared to those who are low on empathy. No directional hypothesis about the possible moderating effects of callous-unemotional traits is made, due to inconsistencies in the literature. The study is conducted in a normative sample and the focus is on the individual characteristics that may contribute to a child being more responsive or more resistant to an anti-bullying intervention, regardless of their past experiences with peer victimization. Therefore, previous involvement with bullying as either a perpetrator or a target is controlled for in the analyses. For the generalizability of the results, gender of the students as well as the teacher were controlled for – one female and one male actor were used to represent teacher in the videos.

## Method

### Participants

Data were collected from a convenience sample of secondary schools (*n* = 3) and combined (primary and secondary grades together) schools (*n* = 4) in Finland. The study has been evaluated and approved by the Ethics Committee for Human Sciences of the University of Turku. Due to the COVID-19 pandemic and the school lockdown that took place in Finland during the spring 2020, the data collection was conducted on three occasions, in February 2020, May 2020 and September-October 2020. In February and September–October the data was collected in schools by the first author and trained research assistants using pen and paper questionnaires, whereas in May the data was collected using digital (online) survey. To recruit participants, school principals were contacted to explain the study and to ask them to invite all Grade 7 students at their school to participate. Parents or guardians of the students received information on the study procedures and data protection, and they were asked for an informed consent for their child to participate. Students who returned a completed parental consent form were eligible to participate in a token lottery (two movie tickets per classroom) regardless of whether they were consented to participate. Only students who received parental consent and provided their own assent participated in the study. The sample consisted of 295 students from 38 classrooms in seven schools divided into 22 test groups (all students who participated online were considered as one test group); 273 of them responded to the pen-and-paper questionnaires at school and 22 of them responded to the online questionnaire at home. Out of the 22 students who responded to the online questionnaire, nine reported that they had not watched the video before moving to the second part of the questionnaire (two of them were the only participants from their classroom), and these cases were excluded from further analyses. According to independent samples t-tests, the remaining online participants did not differ from pen-and-paper participants in the study variables, with the exception of the gender of the teacher in the video (*t*(263) = −15.97, *p* < 0.001). Although half of the video messages were delivered by a female teacher and the other half by a male teacher, the online participants all saw a message presented by the female teacher. Out of the 273 students who responded to the pen-and-paper questionnaires, nine were excluded due to clearly patterned responses on the survey. The final sample consisted of 277 students (129 females, 147 males, and one for whom the information on gender was missing; *M*_age_ = 12.93, SD = 0.49) from 37 classrooms and 22 test groups.

### Procedure

Each classroom was assigned to one of the three experimental conditions (condemning, empathy-raising, and combined message) with the exception of one large classroom where students were randomly divided across the three conditions. To facilitate data collection for the schools, students from several classrooms assigned to the same experimental condition (within the same school) were gathered together to participate in the same test group. Participants completed a short survey before and after watching the video where a teacher delivered the anti-bullying message that corresponded with their condition. The pre-questionnaire consisted of items about demographic information (e.g., age, gender), bullying behavior, victimization, empathy, and callous-unemotional traits. Prior to seeing the video and answering questions about their bullying experiences, participants were provided with a definition of bullying. Before seeing the video, participants were asked to imagine that they had been involved in bullying a peer at school and the teacher had invited them to discuss the situation. They were told to listen carefully, since the video would be played only once. Each group saw one of the six videos, (i.e., one of the three messages delivered by either a male or female teacher). After watching the video, participants’ answered questions about their perception of the extent to the teacher had condemned their bullying behavior or tried to arouse their empathy for the victim. Finally, they were asked how likely they would stop their bullying behavior after such a discussion, if it happened to them in real life. The whole procedure took about an hour.

### Measures

#### Intention to stop hypothetical bullying behavior

Participants’ intention to stop bullying behavior was measured using 6 items: If I had been in this situation and the teacher would have talked to me like this, (a) I would stop bullying the classmate; (b) it would be unlikely that I would bully others in the future because of what the teacher said to me; (c) I would not bully others anymore after this discussion; (d) I would probably continue bullying after this (reverse coded); (e) what the teacher said would very likely influence how I treat others in the future; and (f) the teacher’s words would have a strong impact on my behavior. Answers were given on a 6-point scale ranging from 0 = strongly disagree to 5 = strongly agree. The reliability coefficient McDonald’s omega (see Hayes and Coutts 2020) for these six questions was satisfactory (Ω = 0.84).

#### Message received

There were three conditions, condemning message, empathy-raising message and combined message. In the analyses, three dummy-coded variables (1 = participant received this message, 0 = participant did not receive this message) were used to indicate the three conditions, namely, condemning message, empathy-raising message, and combined message.

#### Perceived condemning of the bullying behavior

The extent to which participants felt that the teacher had condemned their hypothetical bullying behavior was measured using 3 items: (a) the teacher clearly mentioned that I have behaved wrongly; (b) the teacher told me that he/she knew that I had been bullying my classmate and demanded that I stop; and (c) the teacher blamed me for the things that have happened. Responses were given on a 6-point scale ranging from 0 = strongly disagree to 5 = strongly agree. Mean scores were calculated for the perceived condemning of the bullying behavior (Ω = 0.86).

#### Perceived empathy-raising

The extent to which participants’ felt that the teacher had tried to arouse their empathy towards the hypothetical ‘victim’ was measured using 4 items: (a) the teacher talked especially about how bad my classmate is feeling; (b) the teacher tried to make me understand how bad my classmate is feeling; (c) the teacher did not blame me, but wanted me to help the classmate who is having a difficult time; and (d) the teacher helped me to understand the difficult situation my classmate is in. Responses were given on a 6-point scale ranging from 0 = strongly disagree to 5 = strongly agree. Mean scores were calculated for the perceived empathy-raising (Ω = 0.79).

#### Affective and cognitive empathy

Students’ level of affective and cognitive empathy was measured using the Basic Empathy Scale (BES; Jolliffe & Farrington, 2006). The BES is a 20-item self-report scale developed to assess both affective (11 items; e.g., “After being with a friend who is sad about something, I usually feel sad”) and cognitive empathy (9 items; e.g., “I am not usually aware of my friend’s feelings;” reverse coded). Answers were given on a 6-point scale ranging from 0 = strongly disagree to 5 = strongly agree. Separate mean scores were calculated for affective (Ω = 0.83) and cognitive empathy (Ω = 0.81).

#### Callous-unemotional traits

Students’ callous-unemotional traits were measured using The Inventory of Callous-Unemotional Traits (ICU; Frick, 2004). The ICU is a 24-item self-report scale used to assess three aspects of callous-unemotional traits in youth: uncaring, callousness, and unemotionality (e.g., “I do not feel remorseful when I do something wrong.”, “The feelings of others are unimportant to me”). Answers were given on a 6-point scale ranging from 0 = strongly disagree to 5 = strongly agree. Mean scores were calculated for the total callous-unemotional traits scale (Ω = 0.86, excluding items 2 and 10 as recommended by Ray et al., 2016; also, in the data used in the current study, these two items reduced, rather than increased, the reliability).

#### Control variables

Control variables used in the analyses were gender of the participant (0 = girl, 1 = boy), the teacher (0 = female, 1 = male), and self-reported frequency of bullying and victimization. The gender of the students was taken into account because previous studies indicate that there are gender differences in bullying behavior (Cook et al., 2010), empathy (Farrell & Vaillancourt, 2021) and callous-unemotional traits (Essau et al., 2006). The self-reported frequency of bullying and victimization were measured using the global single items of bullying and victimization from the revised Olweus’s Bully/Victim Questionnaire (Olweus, 1996). Before responding the participants were provided with the definition of bullying (Olweus, 1996). Responses to the questions “How often have you bullied others at school in the last couple of months?” and “How often have you been bullied at school in the last couple of months?” were given on a 5-point scale (0 = not at all, 1 = only once or twice, 2 = two or three times a month, 3 = about once a week, and 4 = several times a week). The global items have been shown to be valid measures of bullying and victimization (Olweus & Limber, 2019; Salmivalli et al., 2011; Solberg & Olweus, 2003).

### Analysis Plan

First, to ensure that the messages were perceived as intended (validity check), regression analyses were conducted to test the effects of the different messages on perceived condemning of the bullying behavior and perceived empathy-raising. The teacher speaking in the video, gender of the participant, and self-reported frequency of bullying and victimization were included in the analysis as control variables. Second, a series of hierarchical regression analyses were conducted with intention to stop bullying as the dependent variable. In the first step, after entering the control variables, the main effects of the type of message received on students’ intention to stop bullying were tested (Model 1). Separate models were run for empathy and callous-unemotional traits. In the second step of each model, the main effects of affective and cognitive empathy (Model 2a), and callous-unemotional traits (Model 3a) were added. In the third step, the interactions between the type of message received and affective and cognitive empathy (Model 2b), and callous-unemotional traits (Model 3b) were added. In all analyses, type of message received was included as two dummy-coded variables (condemning message vs. not, empathy-raising message vs. not). Thus, combined message was the reference category. Also, affective and cognitive empathy, and callous-unemotional traits were grand mean centered. The analyses were conducted using *M*plus 8 (Muthén and Muthén 1998–2021) and the robust version of maximum likelihood estimation (MLR). Missing data was handled using full information maximum likelihood estimation (FIML). The differences between the test groups were accounted for by using the COMPLEX option in *M*plus which corrects distortions in standard error estimates caused by the clustering of observations (i.e., between-level variations). Test group was chosen as the clustering variable in order to control for situational factors in the testing session that might make the responses of students within test groups more similar to each other. The ICC for test group for intention to stop bullying was 0.04, indicating that 4% of the total variance was due to differences between the test groups. Significant interactions were probed using the Pick-a-Point Approach where the model was run three times so that the significant moderator was centered around its mean, a standard deviation below the mean and a standard deviation above the mean (Hayes & Montoya, 2017).

## Results

### Descriptive Statistics

The correlations and descriptive statistics of the study variables are presented in Table 1. As expected, perceived condemning of the bullying behavior was positively correlated with seeing the condemning message (*r* = 0.39) and the combined message (*r* = 0.25), and negatively correlated with seeing the empathy-raising message (*r* = −0.64). Similarly, perceived empathy-raising was positively correlated with seeing the empathy-raising message (*r* = 0.43), and negatively correlated with seeing the condemning message (*r* = −0.54). Intention to stop bullying was positively correlated with both affective empathy (*r* = 0.30) and cognitive empathy (*r* = 0.34), and negatively correlated with self-reported bullying (*r* = −0.40) and callous-unemotional traits (*r* = −0.53). Intention to stop bullying was lower among boys compared to girls (*r* = −0.20). Also, boys tended to score higher than girls in callous-unemotional traits (*r* = 0.22) and lower in both affective (*r* = −0.48) and cognitive empathy (*r* = −0.33). Self-reported bullying was positively correlated with self-reported victimization (*r* = 0.24). Callous-unemotional traits were positively correlated with self-reported bullying (*r* = 0.23) and negatively correlated with both affective (*r* = −0.44) and cognitive empathy (*r* = −0.45). Affective empathy was positively correlated with self-reported victimization (*r* = 0.13) and affective and cognitive empathy were both negatively correlated with self-reported bullying (*r* = −0.12 and *r* = −0.14), and the two types of empathy were positively correlated with each other (*r* = 0.42).

**Table 1:** Correlations and descriptive statistics of study variables

Variable	1	2	3	4	5	6	7	8	9	10	11	12	*13*
1. Intention to Stop Bullying	–												
2. Perceived Condemning	0.09	–											
3. Perceived Empathy-raising	0.29***	−0.27***	–										
4. Teacher (male)	−0.10	−0.04	0.05	–									
5. Boy	−0.20***	−0.08	0.08	−0.05	–								
6. Bullying	−0.40***	0.06	−0.02	0.05	0.12*	–							
7. Victimization	0.00	−0.03	0.08	0.12	−0.04	0.24***	–						
8. Condemning	−0.10	0.39***	−0.54***	−0.02	−0.10	0.02	−0.08	–					
9. Empathy-raising	0.03	−0.64***	0.43***	0.02	0.04	−0.09	0.02	−0.52***	–				
10. Combined	0.07	0.25***	0.12	−0.00	0.07	0.07	0.07	−0.49***	−0.49***	–			
11. Affective empathy	0.30***	0.09	0.04	0.00	−0.48***	−0.12*	0.13*	0.03	−0.01	−0.02	–		
12. Cognitive empathy	0.34***	0.02	0.16**	−0.05	−0.33***	−0.14*	0.07	−0.00	−0.03	0.04	0.42***	–	
13. CU traits	−0.53***	−0.07	−0.21***	0.06	0.22***	0.23***	−0.05	0.11	−0.02	−0.09	−0.44***	−0.45***	–
*M*	4.15	3.60	3.51	0.48	0.53	0.13	0.34	0.34	0.34	0.31	2.82	3.75	1.52
*SD*	0.91	1.45	1.16			0.41	0.84				0.86	0.64	0.63
Min	0.83	0.00	0.00			0.00	0.00				0.64	2.11	0.00
Max	5.00	5.00	5.00			3.00	4.00				4.91	5.00	3.36

*Note: N* = 277. Correlations coefficients between binary variables are phi coefficients

***p < 0.001. **p < 0.01. *p < 0.05

The mean of the intention to stop bullying was 4.15 (scale 0–5). This means that overall, the intention to stop bullying was quite high across the different messages received, at least right after hearing the message.

### Perceptions of the Condemning, Empathy-Raising, and Combined Messages

In the first model of the validation analysis, student-perceived condemning of the bullying behavior was predicted and in the second model student-perceived empathy-raising was predicted. The type of message received was included in the analysis as main predictor and the teacher, gender of the student and self-reported bullying and victimization were included in the analysis as control variables. The first model explained 41.7 % of the variance in the perceived condemning of the bullying behavior and the second model explained 32.6 % of the variance in the perceived empathy-raising. The condemning message was perceived as equally condemning (*b* = 0.22, *SE* = 0.16, *CI* = −0.09, 0.53, *p* = 0.159) and as less empathy-raising (*b* = −1.05, *SE* = 0.16, *CI* = −1.36, −0.74, *p* < 0.001) than the combined message. The empathy-raising message was perceived as less condemning than the combined message (*b* = −1.82, *SE* = 0.17, *CI* = −2.14, −1.50, *p* < 0.001), and more empathy-raising than the combined message (*b* = 0.49, *SE* = 0.15, *CI* = 0.21, 0.78, *p* = 0.001).

### Intention to Stop Bullying

#### Main effects of messages

In the first model, the main effects of the different messages received on intention to stop bullying were tested, after controlling for teacher, gender of the students, self-reported bullying and victimization (Model 1, see Table 2). Together, these variables explained 21.2 % of the variance in the intention to stop bullying. Intention to stop bullying was significantly lower after viewing the male (*M* = 4.05, *SD* = 0.96), as compared with the female teacher (*M* = 4.24, *SD* = 0.86; *b* = −0.18, *SE* = 0.08, *CI* = −0.35, −0.02, *p* = 0.032). The intention to stop bullying was significantly lower for boys (*M* = 3.97, *SD* = 0.98) than girls (*M* = 4.34, *SD* = 0.78; *b* = −0.30, *SE* = 0.10, *CI* = −0.50, −0.11, *p* = 0.003). Self-reported frequency of bullying was negatively related to intention to stop bullying (*b* = −0.90, *SE* = 0.11, *CI* = −1.12, −0.67, *p* < 0.001). However, self-reported frequency of victimization was not significantly related to intention to stop bullying. Regarding the different messages received, the effect of the empathy-raising message (*M* = 4.18, *SD* = 0.82) on students’ intention to stop bullying did not significantly differ from the effect of the combined message (*M* = 4.23, *SD* = 0.86). However, participants’ intention to stop bullying was significantly lower for those who received the condemning message (*M* = 4.04, *SD* = 1.03) than for those who received the combined message (*b* = −0.25, *SE* = 0.11, *CI* = −0.47, −0.03, *p* = 0.026).

**Table 2:** Hierarchical regression for predicting intention to stop bullying

	Model 1				Model 2a				Model 2b			
Variable	*b*	95 % *CI*	*SE*	*p*	*b*	95 % *CI*	*SE*	*p*	*b*	95 % *CI*	*SE*	*p*
Teacher (male)	−0.18	[−0.35, −0.02]	0.08	0.032	−0.15	[−0.34, 0.04]	0.10	0.126	−0.14	[−0.32, 0.05]	0.10	0.151
Boy	−0.30	[−0.50, −0.11]	0.10	0.003	−0.05	[−0.29, 0.19]	0.12	0.683	−0.05	[−0.28, 0.19]	0.12	0.701
Bullying	−0.90	[−1.12, −0.67]	0.11	0.000	−0.81	[−1.03, −0.58]	0.11	0.000	−0.84	[−1.07, −0.60]	0.12	0.000
Victimization	0.10	[−0.01, 0.21]	0.05	0.063	0.06	[−0.05, 0.17]	0.06	0.305	0.08	[−0.03, 0.18]	0.05	0.149
Condemning	−0.25	[−0.47, −0.03]	0.11	0.026	−0.23	[−0.48, 0.02]	0.13	0.075	−0.23	[−0.47, 0.02]	0.12	0.066
Empathy-raising	−0.13	[−0.33, 0.07]	0.10	0.205	−0.11	[−0.34, 0.11]	0.11	0.321	−0.12	[−0.33, 0.09]	0.11	0.260
Affective empathy					0.16	[0.03, 0.30]	0.07	0.019	0.09	[−0.09, 0.27]	0.09	0.323
Cognitive empathy					0.30	[0.14, 0.47]	0.09	0.000	0.14	[−0.14, 0.42]	0.14	0.334
Condemning x Affective empathy									0.04	[−0.26, 0.33]	0.15	0.817
Empathy-raising x Affective empathy									0.19	[−0.05, 0.43]	0.12	0.121
Condemning x Cognitive empathy									0.41	[0.11, 0.72]	0.16	0.009
Empathy-raising x Cognitive empathy									0.04	[−0.29, 0.38]	0.17	0.801
*R*^2^	0.212		0.06	0.000	0.286		0.05	0.000	0.307		0.06	0.000

*Note: N* = 277. Reference categories are female, girl and the combined message

#### Main effects of student characteristics

Regarding the main effects of empathy on intention to stop bullying (Model 2a), including affective and cognitive empathy traits to Model 1 explained an additional 7.4% of the variance in the outcome variable. Both, affective empathy (*b* = 0.16, *SE* = 0.07, *CI* = 0.03, 0.30, *p* = 0.019), and cognitive empathy (*b* = 0.30, *SE* = 0.09, *CI* = 0.14, 0.47, *p* < 0.001) were positively related to intention to stop bullying.

For the model examining the effect of callous-unemotional traits on intention to stop bullying (Model 3a), adding this predictor to Model 1 explained additional 16.7 % of the variance in the intention to stop bullying. Callous-unemotional traits were negatively related to intention to stop (*b* = −0.63, *SE* = 0.08 *CI* = −0.78, −0.48, *p* < 0.001).

#### Interaction effects

**Fig. 1:**
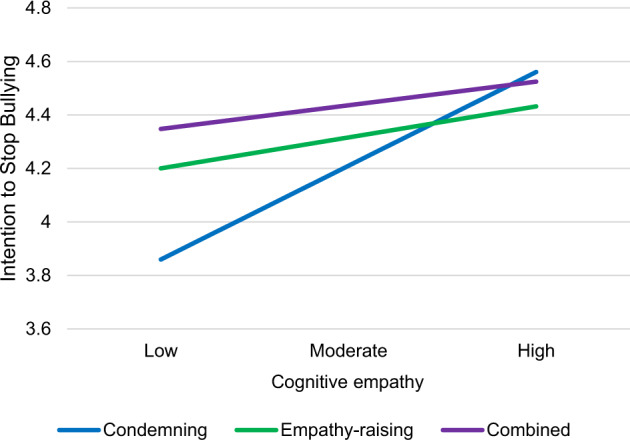
Intention to stop bullying for different messages received on low, moderate and high values of cognitive empathy. Fitted lines reflect all other variables at value 0 (i.e., girls at average level of affective empathy, who have not bullied or been victimized, and who saw the different messages presented by female teacher)

In Model 2b, the interaction terms between the different messages received and affective and cognitive empathy (Table 2) were added to Model 2a. The interactions explained additional 2.1% of the variance in the intention to stop bullying. Affective empathy did not moderate the effects of different messages on intention to stop bullying. However, cognitive empathy moderated the effects of the condemning message (*b* = 0.41, *SE* = 0.16, *CI* = 0.11, 0.72, *p* = 0.009) but not the effects of the empathy-raising message on intention to stop bullying. Examination of the simple slopes (Fig. 1.) revealed that cognitive empathy had a positive effect on intention to stop bullying among those who received the condemning message (*b* = 0.55, *SE* = 0.09, *CI* = 0.37, 0.73, *p* < 0.001) but not among those who received the empathy-raising message (*b* = 0.18, *SE* = 0.10, *CI* = −0.01, 0.37, *p* = 0.060) or the combined message (*b* = 0.14, *SE* = 0.14, *CI* = −0.14, 0.42, *p* = 0.334). Probing the interaction showed that, among students low in cognitive empathy, intention to stop bullying was significantly lower for those who saw the condemning message compared to those who saw the combined message (*b* = −0.49, *SE* = 0.19, *CI* = −0.86, −0.12, *p* = 0.010). However, among students moderate (*b* = −0.23, *SE* = 0.12, *CI* = −0.47, 0.02, *p* = 0.066), and high in cognitive empathy (*b* = 0.04, *SE* = 0.12, *CI* = −0.20, 0.27, *p* = 0.762), intention to stop bullying did not differ between the condemning message compared to the combined message.

When the interaction terms between the different messages received and callous-unemotional traits (Model 3b) were added to Model 3a, the interactions explained additional 0.2% of the variance in the intention to stop bullying. Interactions between callous-unemotional traits and the different messages were not significant (Table 3).

**Table 3:** Hierarchical regression for predicting intention to stop bullying

	Model 3a				Model 3b			
Variable	*b*	95 % *CI*	*SE*	*p*	*b*	95 % *CI*	*SE*	*p*
Teacher (male)	−0.13	[−0.31, 0.05]	0.09	0.165	−0.13	[−0.31, 0.05]	0.09	0.151
Boy	−0.13	[−0.29, 0.02]	0.08	0.087	−0.14	[−0.29, 0.01]	0.08	0.061
Bullying	−0.67	[−0.86, −0.48]	0.10	0.000	−0.67	[−0.89, −0.46]	0.11	0.000
Victimization	0.06	[−0.04, 0.15]	0.05	0.222	0.06	[−0.03, 0.15]	0.05	0.177
Condemning	−0.13	[−0.34, 0.09]	0.11	0.245	−0.13	[−0.34, 0.08]	0.11	0.233
Empathy-raising	−0.08	[−0.26, 0.11]	0.09	0.422	−0.08	[−0.27, 0.10]	0.10	0.377
CU traits	−0.63	[−0.78, −0.48]	0.08	0.000	−0.52	[−0.74, −0.31]	0.11	0.000
Condemning x CU traits					−0.16	[−0.47, 0.15]	0.16	0.298
Empathy-raising x CU traits					−0.11	[−0.37, 0.14]	0.13	0.386
*R*^2^	0.379		0.05	0.000	0.381		0.05	0.000

*Note: N* = 277. Reference categories are female, girl and the combined message

## Discussion

Knowing which strategy will be most effective when intervening in cases of bullying is important for teachers, especially when the case involves adolescent perpetrators. Indeed, tackling school bullying has been found to be more challenging with adolescents than with children (Yeager et al., 2015), possibly because of decreased obedience to adult authority and a stronger desire for status among peers in this developmental period (see Salmivalli et al., 2021). Previous research on this topic indicates that condemning the bullying behavior and trying to raise empathy for the victim are both effective strategies. However, in these previous studies, the exact messages that teachers delivered to the perpetrators were either not known or could not be controlled for in the study design. To address these limitations, the present study used an experimental design with video vignettes depicting a teacher talking to a student (the participant) after they had hypothetically bullied a peer. The effects of hearing a message that either condemned their behavior (confronting approach), attempted to raise their empathy for the victim (non-confronting approach), or did both on students’ intention to stop bullying were examined.

The first main objective of the study was to test the impact of different messages (condemning, empathy-raising, and combined message) on students’ intention to stop bullying. As expected, the students’ intention to stop bullying was on average equally high for those who saw the condemning or empathy-raising message. This finding is in line with previous studies that found no evidence that either the confronting or non-confronting approach was overall more effective than the other (Garandeau et al., 2014b; Garandeau et al., 2016; Johander et al., 2021). However, as it was also hypothesized, the students’ intention to stop bullying was highest for those who saw the combined message. Thus, this study adds to previous findings, providing further evidence that combining elements from both approaches might be more effective than using a single approach (Garandeau et al., 2016).

The second objective of this study was to examine whether affective and cognitive empathy, and callous-unemotional traits have an influence on students’ intention to stop bullying. The results were also in line with expectations. Both affective and cognitive empathy positively predicted, and callous-unemotional traits negatively predicted students’ intention to stop bullying. Callous-unemotional traits explained more variance in intention to stop than affective and cognitive empathy. These results suggest that youth with higher levels of empathy are more likely to stop (or at least intend to) their bullying behavior in response to adult’s targeted intervention, not only less likely to bully others as indicated by previous studies (e.g., van Noorden et al., 2015). The opposite seems to be true for youth with higher levels of callous-unemotional traits; they are more likely to bully others (Zych et al., 2019), but also less likely to respond to an intervention aimed at decreasing their bullying behavior.

Finally, whether the effects of the different messages on students’ intention to stop bullying varied depending on their level of empathy or callous-unemotional traits was also examined. Contrary to what was expected, affective empathy did not moderate the relative effectiveness of the different messages. Also, callous-unemotional traits did not moderate the relative effectiveness of the different messages. This means that regardless of the students’ level of affective empathy or callous-unemotional traits, all messages worked equally well (or equally poorly). This, and the lower intention to stop bullying behavior for students high in callous-unemotional traits, is in line with studies suggesting that neither confronting or non-confronting interventions are likely to work with youth high on callous-unemotional traits (Viding et al., 2009).

In contrast, youth’s ability to take the perspective of others (i.e., cognitive empathy) was found to moderate the relative effectiveness of the condemning message. Adults who used a condemning message were the least effective at encouraging youth with lower cognitive empathy to change their behavior, whereas all the messages were equally effective among youth with high cognitive empathy. This finding indicates that students with lower levels of cognitive empathy would not be expected to be as responsive to a condemning message compared to an empathy-raising, or a combined message. Previous research has found that bullying perpetrators do not form a homogeneous group (e.g., Peeters et al., 2010). Whereas some bullying perpetrators might be cold manipulators with superior theory-of-mind skills (Renouf et al., 2010; Sutton et al., 1999), some might engage in bullying partly because of their deficiencies in their ability to take the perspective of others (Monks et al., 2005). For this group of perpetrators low in cognitive empathy, raising their awareness of the victims’ suffering might be more effective than just condemning their behavior. Since for many students the content of the message does not matter, and simply condemning the behavior is not helpful for those low in cognitive empathy, it might be most efficient to use the combined message with all the students.

Regarding the covariates, victimization was not associated with students’ intention to stop bullying. However, teacher, student gender, and frequency of bullying were associated with intention to change. Intention to stop bullying was lower for those students who saw the messages presented by the male teacher compared to the female teacher. However, since the messages were only delivered by one male and one female, it might be that the observed difference in students’ intention to stop was caused by something other than teacher gender. For example, even though the teachers in the videos were asked to be as neutral as possible, it is possible that there were differences in the intensity with which the teachers talked or in the amount of emotion they expressed. Intention to stop bullying was lower for boys than girls, a finding that is also in line with previous studies. Boys are generally more likely than girls to bully others (Cook et al., 2010). The current study shows that they are also less likely to say that they would stop their bullying behavior as a response to an intervention. Finally, the frequency of bullying negatively predicted students’ intention to stop bullying. Thus, the more the student reported recent bullying behavior towards others in real life, the lower they believed their intention to stop would be after receiving this type of targeted anti-bullying intervention. However, only 11.6% of the students reported that they had bullied others at school in the last couple of months.

### Limitations

Compared to previous studies on the effects of different approaches for targeted interventions, the experimental design of the present study is a clear strength, because the content of the message could be controlled for. However, it also has limitations. First, as described above, most students in the sample reported that they had not bullied others. According to the results, bullies were overall less responsive to the messages. Thus, results of the current study might be different in a sample including only students who actually bully other students in real life.

Second, the study examined students’ intention to stop their hypothetical bullying behavior as a response to a video vignette depicting an adult talking to them. It is unclear, to what extent the obtained results can be generalized to a real-life setting. However, this was a deliberate decision, as the vignettes allowed control over exactly what was said in the different conditions, which had never been done before.

Third, the study focused on the effects of some psychological traits on students’ intention to stop their bullying behavior. However, there are other characteristics, that might have an impact on the effectiveness of the different messages, such as student popularity, which has been shown to moderate the effectiveness of whole anti-bullying programs on bullying reductions (Garandeau et al., 2014a). In addition, the quality of teacher-student relationship, characteristics of the teacher (e.g., warmth, assertiveness), or features of the specific bullying case (such as whether bullying is done by a group or a single person, whether it has been going on for a long time or not) may all play a role in how successful a particular approach is ending the bullying. Future studies should examine the potential moderating effects of these characteristics.

Fourth, this study focused on how the condemning of bullying behavior, the attempts to arouse empathy, and the combination of both are associated with students’ intention to stop their bullying behavior. Most previous studies had also focused only on the effectiveness of the condemning or empathy arousal. However, there are other elements that teachers might add to the discussions, such as student empowerment and student blame. Future studies should examine the effects of other additional factors on students’ intention to stop their bullying behavior or whether they actually stop their bullying behavior in real life. Future studies should also examine other outcomes besides intention to stop bullying, such as how the different approaches affect students’ perception of the teacher and the student-teacher relationship. Finally, due to the COVID-19 pandemic and the lockdown that took place in Finland during the spring 2020, some students participated in the study online. Thus, there was less control over the situation.

### Implications

The study has three main practical implications. First, in light of current evidence (including the present results as well as prior findings), it appears that combining condemning the bullying behavior with attempts at arousing empathy for the victim leads to best outcomes, at least in terms of intention to stop bullying behavior. Therefore, the combined approach seems to be the optimal strategy to tackle cases of bullying, especially when teachers do not know the student characteristics. When they happen to know that the perpetrator lacks in cognitive empathy, it might be preferable to avoid using only condemning of the behavior. Second, findings of the study support the idea that students with high levels of callous-unemotional traits and low levels of empathy are more resistant to adult interventions. This suggests that it is crucially important to follow the situation and make sure that the victimized students’ situation improves. If bullying still continues, further actions need to be taken. Third, it does not seem overall crucial to adjust the intervention strategy to individual characteristics of students, with the exception that those with difficulties understanding others’ perspective may respond less well to hearing that their behavior is not tolerated without also hearing that their behavior is hurtful.

## Conclusion

Empirical investigations comparing the effects of various strategies that teachers may use during discussions with bullying perpetrators are scarce and strongly needed, especially in adolescent populations. This study addressed two main limitations of prior research by using an experimental design which allowed the control and manipulation of the exact content of the teacher messages. It also investigated the possible moderating effect of empathy and callous-unemotional traits on adolescents’ responses to the various strategies. On average, the condemning and the empathy-raising messages appeared to be equally effective at encouraging youth to stop bullying others; however, a combined message led to the highest intention to stop. Regardless of the message used, intention to stop bullying after viewing the video was lower for youth who engage in bullying more frequently, youth high in callous-unemotional traits and youth low in affective and cognitive empathy. The relative effectiveness of the messages did depend on students’ level of cognitive empathy. For those high in cognitive empathy, the type of message did not affect their intention to stop bullying. However, for those low in cognitive empathy, the condemning message was the least likely to lead to intention to stop bullying. These findings further support the idea that it is best to combine clear disapproval of bullying with attempts at raising empathy for the victims and emphasize the necessity for prevention and intervention school-based programs to focus on increasing students’ empathy for others. Further research is encouraged to replicate the finding that a lack of understanding of others’ perspective would be associated with lower responsiveness to adults’ expressed disapprobation of bullying.
